# Utility of shaking chills as a diagnostic sign for bacteremia in adults: a systematic review and meta-analysis

**DOI:** 10.1186/s12916-024-03467-z

**Published:** 2024-06-11

**Authors:** Tetsuro Aita, Hiroaki Nakagawa, Sei Takahashi, Toru Naganuma, Keisuke Anan, Masahiro Banno, Sugihiro Hamaguchi

**Affiliations:** 1 Department of General Internal Medicine, Fukushima Medical University, Fukushima City, 1 Hikarigaoka, Fukushima, 960-1295 Japan; 2 Department of Clinical Epidemiology, Graduate School of Medicine, Fukushima Medical University, Fukushima, Japan; 3 Futaba Emergency and General Medicine Support Center, Fukushima Medical University, Fukushima, Japan; 4 Division of Respiratory Medicine, Saiseikai Kumamoto Hospital, Kumamoto, Japan; 5 Systematic Review Workshop Peer Support Group (SRWS-PSG), Osaka, Japan; 6 Department of Psychiatry, Seichiryo Hospital, Nagoya, Japan

**Keywords:** Bacteremia, Sepsis, Shivering, Chills, Rigor

## Abstract

**Background:**

Accurate prediction of bacteremia is essential for guiding blood culture collection and optimal antibiotic treatment. Shaking chills, defined as a subjective chill sensation with objective body shivering, have been suggested as a potential predictor of bacteremia; however, conflicting findings exist. To address the evidence gap, we conducted a systematic review and meta-analysis of studies to assess the diagnostic accuracy of shaking chills for predicting bacteremia among adult patients.

**Methods:**

We included studies reporting the diagnostic accuracy of shaking chills or chills for bacteremia. Adult patients with suspected bacteremia who underwent at least one set of blood cultures were included. Our main analysis focused on studies that assessed shaking chills. We searched these studies through CENTRAL, MEDLINE, Embase, the World Health Organization ICTRP Search Portal, and ClinicalTrials.gov. Study selection, data extraction, evaluation for risk of bias, and applicability using the QUADAS-2 tool were conducted by two independent investigators. We estimated a summary receiver operating characteristic curve and a summary point of sensitivity and specificity of the index tests, using a hierarchical model and the bivariate model, respectively.

**Results:**

We identified 19 studies with a total of 14,641 patients in which the accuracy of shaking chills was evaluated. The pooled sensitivity and specificity of shaking chills were 0.37 (95% confidence interval [CI], 0.29 to 0.45) and 0.87 (95% CI, 0.83 to 0.90), respectively. Most studies had a low risk of bias in the index test domain and a high risk of bias and a high applicability concern in the patient-selection domain.

**Conclusions:**

Shaking chills are a highly specific but less sensitive predictor of bacteremia. Blood cultures and early initiation of antibiotics should be considered for patients with an episode of shaking chills; however, the absence of shaking chills must not lead to exclusion of bacteremia and early antibiotic treatment.

**Supplementary Information:**

The online version contains supplementary material available at 10.1186/s12916-024-03467-z.

## Background

Bacteremia is a relatively common yet life-threatening condition associated with high mortality rates [1, 2]. Accurate diagnosis of bacteremia is essential for the selection of antibiotics and treatment duration [3]. Therefore, accurate diagnosis and prediction of bacteremia hold significant clinical importance. Blood culture remains the gold standard for diagnosing bacteremia in patients with suspected bacterial infections [3]. However, the diagnosis is delayed since the results of the blood culture are reported after several days, which often presents a dilemma for physicians to initiate empirical antibiotic treatment. Additionally, predicting bacteremia relies primarily on the clinical context, including subjective symptoms such as chills and laboratory test findings [4–6]. Therefore, the identification of objective signs of bacteremia warrants attention to enable the early recognition and accurate prediction of bacteremia.

Shaking chills constitute objective and subjective manifestations characterized by extreme cold sensations leading to stiffness and shivering all over the body, even when covered with a thick blanket [7, 8]. Alternatively, chills represent subjective symptoms that are characterized only by a sensation of coldness without shivering. Thus, shaking chills possibly constitute a more reliable and recognizable sign in patients with suspected bacteremia, especially in cases where patients present with a difficulty in their verbal communication, such as in dementia [9]. Previous studies have shown the predictive value of shaking chills in diagnosing bacteremia [8, 9]; however, a systematic review involving children has indicated conflicting results [10], and most of the original diagnostic studies had small sample sizes. Therefore, there is a need to synthesize these findings to better understand the clinical impact of shaking chills on the prediction of bacteremia in adults.

In this systematic review, we aimed to perform a meta-analysis of studies that report the diagnostic accuracy of shaking chills in addition to chills for predicting bacteremia in adult patients.

## Methods

### PRISMA reporting guidelines

To present our systematic review, we followed the PRISMA-DTA and PRISMA 2020 statements [11, 12]. The PRISMA-DTA checklist has been included as Additional file 1.

### Eligibility criteria

#### Study design

In this review, we included all cohort studies, secondary analyses of randomized clinical trials, and case–control studies, documenting the diagnostic accuracy of shaking chills or chills for bacteremia. We considered studies regardless of their publication status, including published articles, unpublished articles (e.g., articles not found in electronic databases), meeting abstracts, and letters. However, we excluded case series and case reports from our review. To be eligible for inclusion, the studies needed to provide data on the number of true-positive, false-positive, true-negative, and false-negative patients for the index test based on the reference standard. We did not exclude any studies based on language, country of origin, or observation period.

#### Participants

We included patients aged ≥ 15 years with suspected bacteremia who had undergone at least one set of blood cultures, comprising both an aerobic and anaerobic bottle. The presence of fever was not a requirement for inclusion in this review. We excluded patients who were undergoing targeted temperature management, a procedure that can induce shivering by maintaining the body temperature between 33 and 36 °C [13].

#### Index test

The index test used in this study for the main analysis was based on the presence of shaking chills, shivering, or rigors. Additionally, in the sensitivity analysis, we focused on a complaint of chills to assess the impact of the presence or absence of chills on diagnostic accuracy, while also exploring any difference in performance based on the extent of chills experienced by patients. The definitions for these manifestations were determined by the authors of each study. In this review, “all chills” was defined as any sensation of chills, including shaking chills and chills, as described in the original studies.

#### Reference standard

The reference standard utilized in this study was blood cultures. Generally, one set of blood cultures for adults represents one aerobic bottle and one anaerobic bottle, with 8–10 mL of blood typically required for each bottle. Medical personnel practice skin sterilization before venipuncture and obtain a minimum of two sets of blood cultures from each of the two designated sites, such as both arms, before initiating antibiotic therapy. However, in this review, we defined the reference standard as at least one set of blood cultures to enhance the statistical power of this research by increasing the total number of included studies. No restrictions were placed on the bottle types or manufacturers used for blood cultures across the original study facilities.

#### Target condition

The target condition was true bacteremia, which refers to the presence of bacteria in the bloodstream, excluding contamination. Detailed definitions of true bacteremia and contamination were determined by the authors of the original studies. The review did not impose any restrictions on the types of organisms identified in the cultures.

### Information sources and search strategy

For article retrieval, we searched several electronic databases, including the Cochrane Central Register of Controlled Trials (CENTRAL), MEDLINE, and Embase. Additionally, we conducted searches on the World Health Organization International Clinical Trials Platform (ICTRP) Search Portal and ClinicalTrials.gov websites. Non-English language papers were translated and evaluated for potential inclusion. Furthermore, we comprehensively conducted manual searches of the references cited in all the included articles and examined other studies that cited them to gather relevant information for this review. The search strategy employed in this review has been presented in Additional file 2.

### Study selection

In the initial stage, all the identified articles were downloaded and processed using Rayyan (https://www.rayyan.ai/) [14]. Subsequently, two independent reviewers (TA, TN, ST, or HN) screened the titles and abstracts of these articles, following predefined inclusion and exclusion criteria. After this initial screening, the reviewers independently reviewed the full texts of the extracted articles. In cases where an article only provided an abstract or its adherence to the study’s inclusion criteria was unclear, the original author was contacted for clarification. Any disagreements between the two reviewers were resolved through discussion and consensus. If necessary, a third reviewer was consulted to aid the resolution of any conflicting assessments.

### Data extraction

The pre-checked data extraction form underwent amendments based on our pilot review of 10 randomly selected studies. Following this, in studies that met the eligibility criteria, two authors (TA, TN, ST, or HN) independently extracted data on participant demographics, sample size, testing methods, as well as sensitivity and specificity values reported in each study. Any disagreements between the two reviewers were discussed and resolved. In cases where further clarification or missing information was required, the reviewers either consulted a third reviewer or contacted the original authors.

### Risk of bias and applicability

Two independent authors (TA, TN, ST, or HN) assessed the risk of bias and applicability in the included studies using the Quality Assessment of Diagnostic Accuracy Studies 2 (QUADAS-2) tool, which was modified for this review [15]. Any disagreements between the two authors were resolved through discussion. If any conflict persisted despite this discussion, a third reviewer intervened to resolve the disagreement. In cases where there was uncertainty regarding study designs or outcomes that needed to be assessed, the original authors of the respective studies were contacted for clarification. When no response to our inquiry was received, we proceeded to assess the risk of bias and applicability solely relying on the descriptions within the original articles.

### Diagnostic accuracy measures

To evaluate the diagnostic accuracy, we extracted raw data on shaking chills for the main analysis or all chills for sensitivity analysis, as well as bacteremia diagnoses from each included study. These data were then used to construct 2 × 2 tables, which allowed us to calculate the diagnostic values such as sensitivity and specificity. Point estimates and 95% confidence interval (95% CI) were calculated to estimate the diagnostic performance. These results were visually presented using a forest plot.

### Synthesis of results and meta-analysis

A summary receiver operating characteristic (SROC) curve was generated to illustrate the distribution of sensitivity and specificity values observed in the original studies. To estimate an SROC curve that integrates the results, a hierarchical model was used. Additionally, the bivariate model was used to estimate a summary point with 95% CI and 95% prediction interval. Throughout the synthesis of results and meta-analysis, we followed the recommended methods in the Cochrane Handbook for Systematic Reviews of Diagnostic Test Accuracy (v2.0) [16]. All analyses were performed using RevMan 5.4 and Diagnostic Test Accuracy Meta-Analysis v2.01 (https://crsu.shinyapps.io/dta_ma/).

### Evaluation of heterogeneity

We evaluated the heterogeneity visually by using the SROC curves and forest plots and considered conducting subgroup analyses to investigate the diagnostic accuracy specifically in the older population, those with malignancy, individuals on steroids or immunosuppressive agents, or those who had received antibiotic use prior to blood culture sample collection. However, the included studies did not report the stratified results for these particular subgroups. Thus, rather than performing a subgroup analysis for the prespecified populations, we presented the percentage of these covariates using study-level summaries [17].

Moreover, to examine other potential sources of heterogeneity, we performed additional subgroup analyses on the following categories:

1. The setting of patient enrollment [emergency department (ED) vs. any setting including outpatient department, ward, intensive care unit, and ED]
2. Hemodialysis status [exclusively patients undergoing hemodialysis (HD) vs. others]

We calculated summary estimates of the sensitivity and specificity for the different subgroups by the bivariate model and examined the difference of these values between subgroups through meta-regression using Stata 18.0 [16].

### Sensitivity analysis

In sensitivity analysis, we evaluated the diagnostic accuracy for bacteremia by incorporating an alternative index test that considered all chills. Furthermore, although not pre-specified, we conducted an assessment of diagnostic accuracy exclusively within studies that included patients with blood culture contamination, considering them within the non-bacteremia group during the analysis. This approach was adopted to prevent potential biases and ensure a more accurate estimation.

### Evaluation of publication bias

We searched clinical trial registries (ClinicalTrials.gov and ICTRP) for completed but unpublished studies. Statistical tests were not performed according to the Cochrane Handbook [16].

### Protocol publication and the difference between protocol and review

We registered the protocol for this review in PROSPERO (CRD42021282466). Although our initial plan was to assess heterogeneity by investigating potential differences in sensitivity, specificity, or both results among various subgroups (such as different age categories, individuals with malignancy, those taking immunosuppressants, and those who had received antibiotics prior to blood culture collection), we were unable to conduct this evaluation due to insufficient data on these subgroups. Alternatively, we reported the proportion of these subgroups presented in each included study. Furthermore, for the same reason, we could not conduct a sensitivity analysis on the diagnostic accuracy of shaking chills for patients who received antibiotics before the collection of blood cultures. We implemented further subgroup analyses and sensitivity analyses based on covariates and scenarios that were not predefined, as mentioned previously.

## Results

### Study selection

**Fig. 1:**
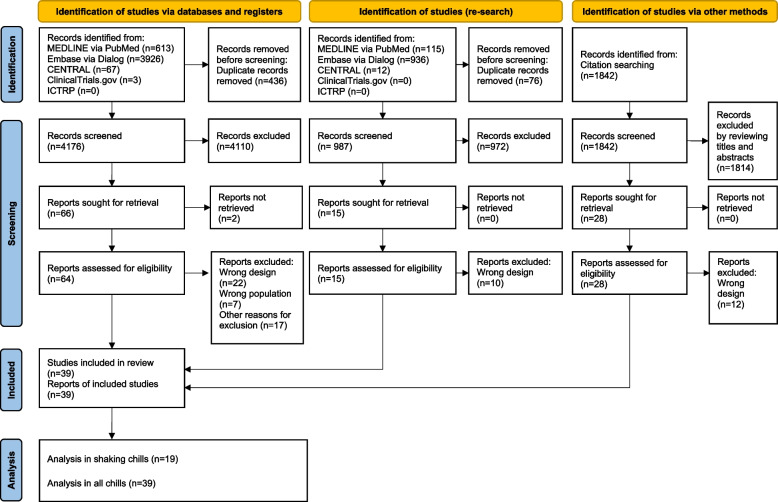
PRISMA 2020 flow diagram. We re-searched the database, articles citing included studies, and those cited in the included research twice. In the flow of database re-search and citation search, the total number of articles based on the additional searches is described. CENTRAL, Cochrane Central Register of Controlled Trials; ICTRP, International Clinical Trials Registry Platform; RCTs, randomized controlled trials

We retrieved 728 articles from MEDLINE, 4862 articles from Embase, 79 articles from CENTRAL, 3 articles from ClinicalTrial.gov, and 1842 from citation searching. After screening 107 articles for eligibility, we included 19 articles for the main analysis and 39 articles for the sensitivity analysis. The detailed review process is depicted in Fig. 1, which comprises a flow diagram illustrating the selection and inclusion of the articles.

### Characteristics of included studies

A total of 14,641 patients from 19 studies were included, utilizing shaking chills as the index test. The mean age in these studies varied between 50 and 84 years. These studies were conducted between 1990 and 2023 across multiple countries, including the USA, Australia, European, and Asian countries. Among the 19 studies, 11 had a prospective design, whereas 8 were retrospective. The primary sites for patient enrollment were ED in 7 studies and any hospital setting, including wards, in 12 studies. Patients with contaminated blood cultures from 15 studies were included in the non-bacteremia group for the main analysis. Further information can be found in Table 1 and Additional file 3: Table S1 [4, 7–9, 18–32] (Table S1 includes other study characteristics regarding the incubation time of blood cultures, administered antibiotics before taking blood cultures, severity of patient’s conditions, and patient’s comorbidities). The characteristics of the included studies that focused on chills are presented in Additional file 3: Table S2 [5, 33–51].

**Table 1:** Characteristics of the included studies that used shaking chills as the index test

Author (location)	Year	Design	Enrollment	Inclusion criteria	Exclusion criteria	Number of analyzed patients	Age, years (mean, SD)	Index test	Reference standard (*n* of sets)	Handling of contamination in blood culture (definition, *n* of patients, and analysis)^a^
Bahagon (Israel, single-center) [18]	2007	Retrospective	Consecutive	Patients aged > 16 years who were admitted to the ED with a positive urine culture, clinical symptoms and/or signs of UTI, and at least one blood culture	ND	350	74 (20)	Shaking chills	Blood cultures (≥ 1)	No, unclear, included
Bates (the USA, multicenter) [19]	1997	Prospective	Consecutive	All adult patients, including those admitted in ICU, who underwent blood cultures	ND	881	59 (17)	Shaking chills	Blood cultures (unclear)	No, unclear, included
Bates (the USA, single-center) [20]	1990	Prospective	Consecutive	Patients aged > 15 years who underwent blood cultures during two periods	Having an episode beginning on a date when no patients were enrolled (weekends, holidays)	1007 blood culture episodes (809 patients)	50 (20)	Shaking chills	Blood cultures (≥ 2)	Yes, 81, included
Chassagne (France, multicenter) [21]	1996	Prospective	Consecutive	Older patients aged > 65 years who underwent blood cultures for clinical signs of sepsis, either at admission or during hospitalization	Contamination, HIV-1 infection, previously receiving antibiotics	258	81 (ND)	Shaking chills	Blood cultures (3)	Yes, unclear, excluded
Fujii (Japan, multicenter) [4]	2022	Prospective	Consecutive	Patients aged ≥ 18 years who underwent at least two sets of blood cultures within 24 h of admission	Transfer to another hospital within 24 h of admission, not evaluating patients’ level of gestalt for bacteremia	2014	81 (15)	Shaking chills, chills	Blood cultures (≥ 2)	Yes, unclear, included
Holmqvist (Sweden, Switzerland, Canada, multicenter) [22]	2020	Prospective	ND	Patients aged ≥ 18 years with abnormal vital signs at the ED visit	Missing data on chills, missing diagnoses, failure to ask about the presence of shaking chills	197	71 (17)	Shaking chills	Blood cultures (≥ 1)	Yes, 0, included
Hoogendoorn (Dutch, multicenter) [23]	2002	Prospective	Consecutive	Patients aged ≥ 18 years who underwent two blood cultures for fever at the ED	ND	764	66 (23)	Shaking chills	Blood cultures (unclear)	No, 0, included
Komatsu (Japan, multicenter) [24]	2017	Prospective	Consecutive	Adult patients who underwent blood cultures and were hospitalized	Gastrointestinal diseases, receiving chemotherapy, TPN or PPN, NPO, tube feeding	1847	69 (17)	Shaking chills, moderate chills, mild chills	Blood cultures (unclear)	Yes, 96, excluded
Lee (Taiwan, single-center) [7]	2012	Prospective	Consecutive	Patients aged ≥ 18 years who visited the ED because of fever (> 38.0 °C) within 1 week	Altered consciousness level, no verbal responses, hospitalization within 30 days preceding admission, living in nursing facilities, fungemia, mycobacteremia	396	54 (ND)	Rigors, chills	Blood cultures (2)	Yes, 8, excluded
McNab (Australia, single-center) [25]	2023	Retrospective	Consecutive	Patients aged > 16 years who had blood cultures collected in the ED	Clinical notes were incomplete, unable to be located, or who died in the ED before the significance of the culture could be determined	1857	63 (range 43–80)	Shivering	Blood cultures (unclear)	Yes, 73, included
Pfitzenmeyer (Switzerland, single-center) [26]	1995	Prospective	Consecutive	Older patients (women ≥ 62 years and men ≥ 65 years) who underwent blood culture	ND	558 episodes (438 patients)	84 (7)	Rigors	Blood cultures (≥ 1)	Yes, unclear, included
Sasaki (A)^b^ (Japan, multicenter) [27]	2021	Retrospective	Consecutive	Patients aged ≥ 18 years on maintenance HD with two sets of blood cultures drawn at admission due to suspicion of bacteremia	Inpatients transferred from another hospital, duration of dialysis < 2 months, receiving peritoneal dialysis, receiving HD less than once a week	360	72 (13)	Shaking chills	Blood cultures (≥ 2)	Yes, 0, included
Sasaki (B)^b^ (Japan, multicenter) [27]	2021	Retrospective	Consecutive	Patients aged ≥ 18 years on maintenance HD with two sets of blood cultures drawn at admission due to suspicion of bacteremia	Inpatients transferred from another hospital, duration of dialysis < 2 months, receiving peritoneal dialysis, receiving HD less than once a week	96	72 (12)	Shaking chills	Blood cultures (≥ 2)	Yes, 0, included
Sasaki (Japan, multicenter) [28]	2017	Retrospective	Consecutive	Patients aged ≥ 18 years on maintenance HD who visited the outpatient department or the ED and underwent two sets of blood cultures drawn at initial arrival	Low frequency of HD, peritoneal dialysis, < 2 weeks from the introduction of HD	293	74 (11)	Shaking chills	Blood cultures (≥ 2)	Yes, unclear, included
Takada (Japan, multicenter) [29]	2021	Prospective	Consecutive	Patients aged ≥ 18 years who underwent at least two sets of blood cultures within 24 h of admission	Tube feeding, use of glucocorticoid or immunosuppressants	2009	81 (14)	Shaking chills	Blood cultures (≥ 2)	Yes, unclear, included
Takamatsu (Japan, single-center) [30]	2016	Retrospective	Consecutive	Walk-in ED patients aged ≥ 15 years who underwent blood cultures	Transported by ambulance, age < 15 years	170	63 (21)	Shaking chills, chills	Blood cultures (unclear)	Yes, 29, excluded
Taniguchi (Japan, single-center) [9]	2013	Retrospective	Consecutive	Patients aged ≥ 18 years with suspected bacterial infections who were newly admitted to the ID department	Other than bacterial infection, HIV infection	366	79 (19)	Shaking chills	Blood cultures (≥ 2)	No, unclear, included
Taniguchi (Japan, single-center) [31]	2022	Retrospective	Consecutive	Patients aged ≥ 15 years with suspected cellulitis	Nosocomial infection, not diagnosed with cellulitis after admission, unclear diagnosis, discharge from ED, insufficient data	221	76 (15)	Shaking chills	Blood cultures (≥ 2)	Yes, 2, included
Tokuda (Japan, single-center) [8]	2005	Prospective	Consecutive	Patients aged ≥ 15 years who were admitted to the ED because of fever (≥ 38 °C) since < 2 weeks	ND	526	57 (24)	Shaking chills, moderate chills, mild chills	Blood cultures (2)	No, unclear, included
Yoshino (Japan, single-center) [32]	2023	Retrospective	Consecutive	Patients who underwent their first autologous hematopoietic cell transplantation and patients with intensive chemotherapy for acute myeloid leukemia	ND	471	50 (range 15–71)	Shaking chills	Blood cultures (≥ 2)	Yes, unclear, included

*ND* no data, *ED* emergency department, *UTI* urinary tract infection, *NPO* nil per os, *PPN* peripheral parenteral nutrition, *TPN* total parenteral nutrition, *ID* infectious diseases, *HD* hemodialysis, *ICU* intensive care unit

^a^Information on the definition of contamination, the number of patients for whom contamination was detected, and the approach taken to analyze data of patients with contamination in their blood cultures were handled in each study

^b^Two cohorts are presented separately as they were included in a study conducted by Sasaki in 2021

### Assessment results of risk of bias and applicability

**Fig. 2:**
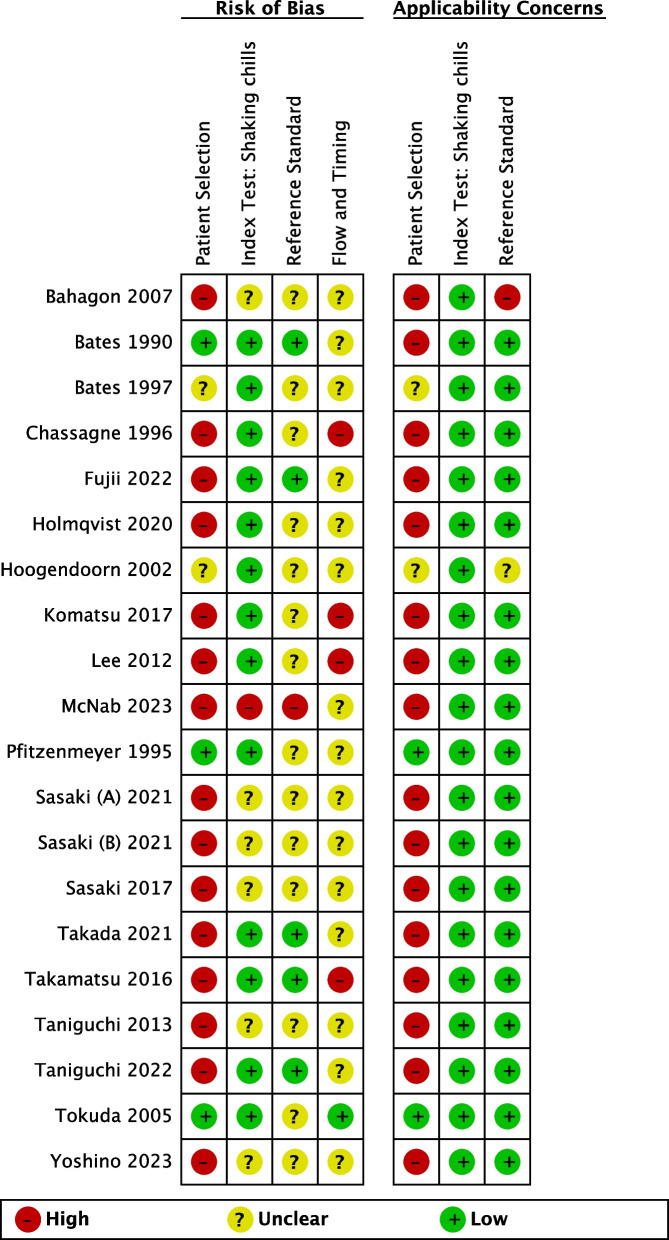
Risk-of-bias assessment of the included studies for shaking chills using the QUADAS-2 tool*. *Two cohorts are presented separately as they were included in a study conducted by Sasaki in 2021

**Fig. 3:**
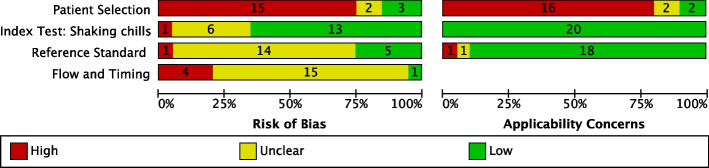
Summary of the QUADAS-2 risk-of-bias assessments in included studies for shaking chills*. *Two cohorts were included in a study conducted by Sasaki in 2021; thus, this figure includes 20 studies although 19 studies were included in the main analysis in our review

The assessment of risk of bias and applicability of the included studies for shaking chills and all chills as the index test have been presented in Figs. 2 and 3 and Additional file 4: Figs. S1 and S2, respectively. As shown in Figs. 2 and 3, 14 studies were deemed to have a high risk of bias in patient selection due to inappropriate exclusion. Although five studies had an unclear risk due to the lack of description of the blinding of blood culture results, the majority of studies showed a low risk of bias in the index test. Regarding the reference standard, the risk of bias was unclear in most studies, except in six, primarily due to the uncertainty of implementation of blinding for the index test results. A total of 18 studies exhibited unclear or high risk of bias in terms of flow and timing, primarily because of inappropriate intervals between the index test and reference standard or the exclusion of patients with blood culture contamination from the analysis. With regard to the applicability, 15 studies generated concern about patient selection, as the study population differed from our target population. However, the majority of studies demonstrated low concern regarding the applicability of the index test and reference standard.

### Results of individual studies and synthesis of the results

**Fig. 4:**
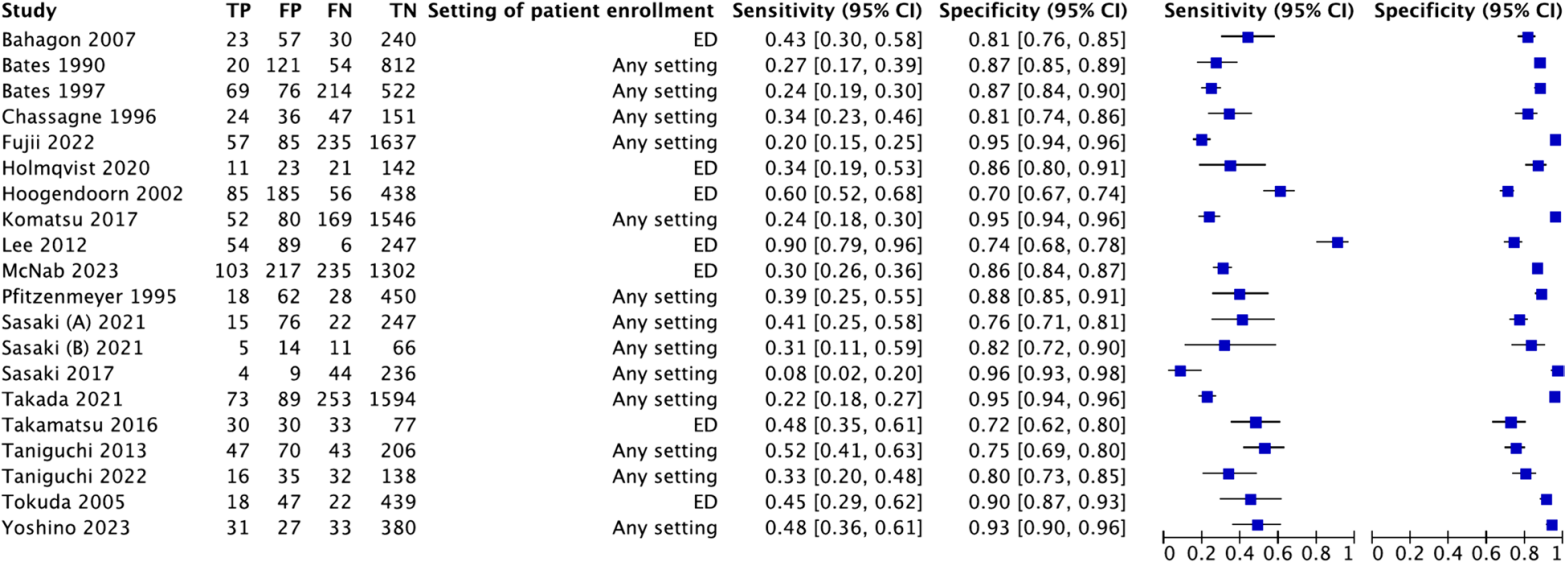
Forest plot of the included studies using shaking chills as the index test. ED, emergency department; any setting: outpatient department, ward, intensive care unit, and ED

**Fig. 5:**
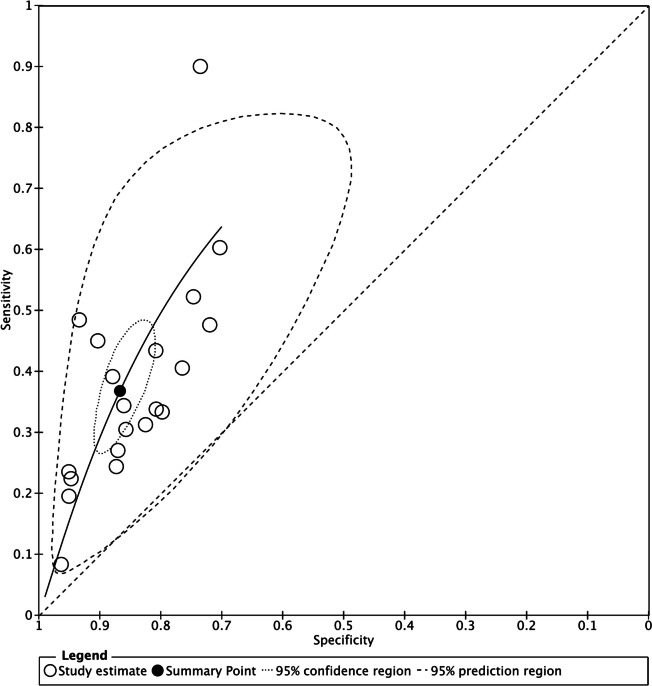
HSROC analysis based on the bivariate model of the included studies for shaking chills. HSROC, hierarchical summary receiver operating characteristic

The diagnostic accuracy measures observed in the included studies for shaking chills and all chills as the index test are shown in Fig. 4 and Additional file 5: Fig. S3, respectively. In Fig. 4, the summary point of specificity demonstrated a narrower range, from approximately 0.8 to 0.9, whereas the sensitivity values showed a wide range. Furthermore, the estimated sensitivity and specificity in all included studies show scattered values (Additional file 5: Fig. S3). Based on the results of the meta-analysis, hierarchical SROC (HSROC) based on the bivariate model of the included studies for shaking chills shows an estimated combined summary point, 95% confidence region, and 95% prediction region. The estimated combined sensitivity and specificity were 0.37 (95% CI, 0.29 to 0.45) and 0.87 (95% CI, 0.83 to 0.90), respectively (Fig. 5). Notably, a visual assessment revealed significant heterogeneity across the included studies.

### Evaluation of heterogeneity

To elucidate the underlying reasons for the notable heterogeneity observed in the meta-analysis, we detailed the characteristics of each study and conducted subgroup analyses. We reported the proportion of specific subgroups within each study, including patients who received antibiotics before blood culture, those with severe conditions, those with comorbidities such as malignancy, diabetes mellitus (DM), and chronic kidney disease (CKD), and those using steroids or immunosuppressants (Additional file 3: Tables S1 and S2). While the severity of patients’ conditions was scarcely reported, the proportion of patients receiving antibiotics before blood cultures varied across the studies, ranging from 5.6 to 37.7%. Furthermore, the proportion of those with comorbidities that could suppress the immune function extremely differed between studies.

Subgroup analysis revealed significant heterogeneity in the patient enrollment setting, while the heterogeneity between studies including only patients undergoing HD and those that did not was not significantly evident (Table 2). On a meta-regression, the absolute differences in sensitivity and specificity between the ED setting and any setting were 0.21 (95% CI, 0.03 to 0.38) and − 0.08 (95% CI, − 0.15 to − 0.01), respectively. By contrast, the absolute differences in sensitivity and specificity between the population on HD and others were − 0.15 (95% CI, − 0.37 to 0.06) and 0.02 (95% CI, − 0.10 to 0.13), respectively.

**Table 2:** Subgroup analysis

Subgroup	Number of studies (*n*)	Participants (*n*)	Sensitivity (95% CI)	Specificity (95% CI)
The setting of patient enrollment				
Limited to emergency department	7	4260	0.51 (0.35–0.67)	0.81 (0.75–0.86)
Any setting (outpatient department, ward, ICU, and ED)	12 (13 cohorts^a^)	10,381	0.30 (0.24–0.37)	0.89 (0.85–0.92)
Hemodialysis status				
Only patients on hemodialysis	2 (3 cohorts^a^)	749	0.23 (0.09–0.48)	0.88 (0.72–0.95)
Others	17	13,892	0.39 (0.31–0.48)	0.86 (0.82–0.90)

*ICU* intensive care unit, *ED* emergency department, *CI* confidence interval

^a^Two cohorts were included in a study conducted by Sasaki in 2021

### Sensitivity analysis

We conducted a sensitivity analysis to assess the diagnostic accuracy of all chills. In a meta-analysis of 39 included studies involving 48,910 individuals, we observed higher estimated combined sensitivity (0.43; 95% CI, 0.35 to 0.52) and lower specificity (0.80; 95% CI, 0.75 to 0.84) for all chills than for shaking chills (Additional file 5: Fig. S4). Furthermore, heterogeneity among the included studies regarding all chills was more prominent than that observed specifically for shaking chills.

Considering the influence of uninterpretable outcomes such as contamination of our findings, we assessed the diagnostic accuracy exclusively within the studies that included patients with blood culture contamination, and we included these patients within the non-bacteremia group during analysis. Consequently, the combined sensitivity and specificity were 0.34 (95% CI, 0.28 to 0.41) and 0.87 (95% CI, 0.83 to 0.91), respectively (Additional file 6: Figs. S5 and S6), and this HSROC suggests a reduced level of heterogeneity compared with that of the main analysis (Additional file 6: Fig. S6). These results suggest that the diagnostic accuracy is consistent with the main analysis. Therefore, our study findings can be considered robust.

## Discussion

### Summary of evidence

In this review, we assessed the diagnostic accuracy of shaking chills for bacteremia by conducting a systematic review and meta-analysis (Table 3). Additionally, we also examined the diagnostic accuracy of all chills in a sensitivity analysis.

**Table 3:** Summary of findings

Patients/populations	Patients aged ≥ 15 years with fever or suspected bacteremia who had at least one set of blood cultures performed; applicable to the review question				
Prior testing	None				
Settings	Patients mainly in the emergency departments, sometimes in the wards; applicable to the review question				
Index test	Shaking chills, shivering, or rigor, which were defined by authors				
Importance	Predicting bacteremia can motivate physicians to collect blood cultures and administer appropriate treatment				
Reference standard	At least one set of blood cultures				
Studies	A total of 19 cohort studies of diagnostic accuracy test were included				
***Test type***	***Summary accuracy (95% CI)***	***No. of participants (studies)***	***Prevalence median (range)***	***Implications***	***Quality and comments***
Shaking chills	Sensitivity 0.37 (0.29–0.45) Specificity 0.87 (0.83–0.90)	14,641 (19)	16% (7–36%)	In a cohort of 100 patients presenting with suspected bacteremia and a prevalence rate of 16%, a history of shaking chills will fail to identify 10 out of 16 patients with bacteremia. Additionally, among the patients without bacteremia, 11 out of 84 individuals would undergo unnecessary blood culture collection	A high risk of bias was observed in patient selection and flow/timing domains due to inappropriate exclusions and inappropriate test intervals/incomplete analysis, respectively. The reference domain had unclear bias due to poor reporting. Fifteen studies raised concerns about the applicability of included patients due to exclusions related to contamination and comorbidities in analysis

Overall, our analysis revealed that shaking chills have emerged as a highly specific manifestation of bacteremia. This finding is consistent with those of previous studies that have assessed the diagnostic accuracy of the different degrees of chills for bacteremia [7–9]. A prospective cohort study reported that the adjusted risk ratio of shaking chills for bacteremia was 12.11 (95% CI, 4.06 to 36.16), followed by moderate chills [4.14 (95% CI, 1.61 to 10.66)] and mild chills [1.77 (95% CI, 0.94 to 3.33)] [8]. Other previous studies showed the adjusted odds ratios of shaking chills for bacteremia were 13.7 (95% CI, 4.47 to 42.0) and 2.53 (95% CI, 1.50 to 4.28) [7, 9]. These studies concluded that the presence of shaking chills helped predict bacteremia. Additionally, although our review demonstrated that shaking chills have lower sensitivity than that of all chills, shaking chills are a more reliable marker for bacteremia because of higher specificity with less variation on comparing both forest plots. This robust finding highlights the increased value of shaking chills.

This is the first systematic review to have assessed the diagnostic accuracy of shaking chills in adult patients with suspected bacteremia, without consideration of underlying illnesses or the patient’s infective state. Although one systematic review focusing on shaking chills as an index test has been reported, this research focused on children and targeted not only bacteremia but also other serious bacterial infections, such as sepsis, meningitis, pneumonia, pyelonephritis, osteomyelitis, septic arthritis, or cellulitis [10]. Thus, while the diagnostic ability of shaking chills had been limited to diagnose these infections without malignancy, shaking chills was helpful in patients with malignancy due to a significantly positive likelihood ratio of 3.47 (95% CI, 2.58 to 4.36). In contrast, our review exclusively concentrated on adult patients with suspected bacteremia, encompassing a diverse array of coexisting medical conditions such as DM (at least 7% of the total cases), CKD (at least 6%), and malignancy (at least 5%). Additionally, the severity of infection-related conditions varied, including some patients requiring intensive care and others experiencing septic shock. Our findings revealed a notable specificity of shaking chills in distinguishing bacteremia, irrespective of the presence of comorbidities or the severity of the infection.

### Clinical implications

The presence of shaking chills can be an indication for obtaining blood cultures, even from patients without severe infection. In clinical practice, discerning the appropriate indications for blood cultures is a challenging task. The failure to detect bacteremia due to lack of blood cultures can lead to fatal consequences. Conversely, obtaining two sets of blood cultures is a relatively invasive and time-consuming procedure that usually involves drawing 36–40 mL of blood from the patient and requiring at least two healthcare professionals for the procedure, who must adhere to strict sterile techniques. Thus, the indications for blood cultures should be carefully determined. To facilitate these decisions, several clinical prediction models for bacteremia have been developed [4–6, 20, 34, 40]. However, some of these models incorporate all chills, rather than just shaking chills, as one of the predictors of bacteremia [4–6, 42]. The complaint of chills is subjective, and obtaining a reliable history of chills is often difficult from patients with communication difficulties, such as those with dementia. However, shaking chills are a more objective and identifiable sign because of visible shivering. Therefore, although shaking chills serve as a highly specific but less sensitive predictor, its presence should prompt consideration for obtaining blood cultures, but blood cultures should not be omitted even in the absence of shaking chills.

The presence of shaking chills can also be an indication of early administration of antibiotics. To use appropriate antibiotics, identification of the source of infection is necessary. Regarding sepsis, the relationship between prompt antimicrobial treatment and clinical outcomes remains controversial [52]. Furthermore, the association between time-to-antibiotics and mortality is limited for patients with sepsis but without shock [53, 54]. However, in case of bacteremia, a > 5-h delay in antibiotic administration is associated with the progression to septic shock in sepsis [55], and a delay of > 12 h is correlated with increased 30-day mortality in bloodstream infections [56]. Therefore, while attempting to investigate the infectious origin, clinicians should sometimes consider prompt administration of broad-spectrum antibiotics even before identifying infectious sites if the patient has an episode of shaking chills.

### Strengths and limitations

The strength of our systematic review is our comprehensive assessment of the diagnostic accuracy across different degrees of chills. Furthermore, this systematic review was conducted with methodological rigor and adherence to established guidelines such as PRISMA 2020, PRISMA-DTA, and the Cochrane Handbook. Moreover, our study exhibits strong external validity, particularly relevant to patients suspected of having bacteremia. Notably, this meta-analysis encompassed a diverse range of infections, such as pneumonia, urinary tract infections, and cellulitis, as it included all adult patients who underwent at least one set of blood cultures. In real-world clinical scenarios, physicians often lack precise information regarding the source of infection when ordering blood cultures for patients. The inclusion of patients with a wide range of infection contexts enhances the generalizability of this study.

Our study has some limitations in terms of data retrieval and the applicability of our research findings. First, the presence of shaking chills may be underreported because this study included eight retrospective studies. In these studies, complaints of shaking chills were extracted from medical records. If medical staff had not reported the complaint regardless of the positive history, the number of complaints of shaking chills would have been underreported, potentially inducing a bias in the estimation of the diagnostic accuracy of shaking chills. Second, concerns regarding the applicability of our results are heightened by the significant heterogeneity observed in our research. This heterogeneity could stem from considerable variation in the study populations, including differences in settings and underlying diseases. Subgroup analysis revealed that shaking chills had a higher sensitivity and slightly lower specificity in patients enrolled via the ED. Although the noticeably high sensitivity observed in the two studies within the ED subgroup [7, 23] may impact the results of this analysis, the diagnostic accuracy of shaking chills could vary across different patient settings. Due to the lack of stratified data, we were unable to comprehensively investigate other sources of heterogeneity. Therefore, further research should aim to narrow the settings and specify the patients’ underlying diseases. Such research, when meta-analyzed, will contribute to improving the applicability of our findings.

## Conclusions

The presence of shaking chills has emerged as a specific but less sensitive predictor of bacteremia. Blood cultures should be considered for patients presenting with shaking chills, even without severe conditions. In addition, timely blood culture collection and early initiation of antibiotics are crucial in cases of shaking chills to prevent septic deterioration. Nevertheless, the lack of shaking chills should not be a reason to dismiss the possibility of bacteremia and the initiation of early antibiotic therapy.

### Supplementary Information

Additional file 1. The PRISMA-DTA checklist.

Additional file 2. Database search strategy.

Additional file 3: Table S1. Other characteristics of the included studies investigating shaking chills. ND, no data; APACHE, acute physiology and chronic health evaluation; SOFA, sequential organ failure assessment; SAPS, simplified acute physiology score; DM, diabetes mellitus; CKD, chronic kidney disease; HD, hemodialysis. *Two cohorts are presented separately as they were included in a study conducted by Sasaki in 2021. Table S2. Characteristics of the included studies investigating chills*. ND, no data; ED, emergency department; UTI, urinary tract infection; CAP, community-acquired pneumonia; ICU, intensive care unit; HIV, human immunodeficiency virus; AIDS, acquired immunodeficiency syndrome; CKD, chronic kidney disease; ESRD, end-stage renal disease; DM, diabetes mellitus; HD, hemodialysis; FUO, fever of unknown origin; APACHE, acute physiology and chronic health evaluation; SOFA, sequential organ failure assessment; PSI, pneumonia severity index; NEWS, national early warning score. *Information on the definition of contamination, the number of patients for whom contamination was detected, and the approach taken to analyze data of patients with contamination in their blood cultures were handled in each study. †If the original articles did not report the standard deviation for age, the age range was specified.

Additional file 4: Fig. S1. Risk-of-bias assessment of the included studies for all chills using the QUADAS-2 tool*. *Two cohorts are presented separately as they were included in a study conducted by Sasaki in 2021. Fig. S2. Summary of the QUADAS-2 risk-of-bias assessments in included studies for all chills*. *Two cohorts were included in a study conducted by Sasaki in 2021. This accounts for the total of 40 studies in this figure, although 39 studies were incorporated in our review.

Additional file 5: Fig. S3. Forest plot of all the included studies using all chills as the index test. Fig. S4. HSROC analysis based on the bivariate model of all the included studies using all chills. HSROC, hierarchical summary receiver operating characteristic.

Additional file 6: Fig. S5. Forest plot of studies analyzed for patients with suspected bacteremia, including those with contaminated blood cultures. Fig. S6. HSROC analysis based on the bivariate model of studies analyzed for patients with suspected bacteremia, including those with contaminated blood cultures. HSROC, hierarchical summary receiver operating characteristic.

## Data Availability

The review protocol was registered in PROSPERO (CRD42021282466). All data were extracted from previously published studies. PRISMA-DTA checklist, search strategy, and all study characteristics were described in this study and additional files.

## Abbreviations

CENTRAL: Cochrane Central Register of Controlled Trials
CKD: Chronic kidney disease
DM: Diabetes mellitus
ED: Emergency department
HSROC: Hierarchical summary receiver operating characteristic
ICTRP: International Clinical Trials Platform
QUADAS-2: Quality Assessment of Diagnostic Accuracy Studies 2
