# Supplementary material for: Utility of shaking chills as a diagnostic sign for bacteremia in adults: a systematic review and meta-analysis
Source: BMC Med. 2024 Jun 11;22:240. doi: 10.1186/s12916-024-03467-z (PMC11167933; doi:10.1186/s12916-024-03467-z)
Supplement: Supplementary file 2 — Additional file 2. Database search strategy. [file 12916_2024_3467_MOESM2_ESM.docx]

Additional file 2: Database search strategy

**CENTRAL search strategy**

#1 [mh bacteremia]

#2 [mh sepsis]

#3 ("bloodstream" NEXT infection*):ti,ab

#4 Bacteremia:ti,ab

#5 Sepsis:ti,ab

#6 pyemia:ti,ab

#7 #1 OR #2 OR #3 OR #4 OR #5 OR #6

#8 [mh chills]

#9 [mh shivering]

#10 Shivering:ti,ab

#11 Chill*:ti,ab

#12 rigor:ti,ab

#13 shaking:ti,ab

#14 #8 OR #9 OR #10 OR #11 OR #12 OR #13

#15 #7 AND #14

#16 ([mh animals]) NOT ([mh humans])

#17 #15 NOT #16

Hits = 67 (20/10/2021)

#1 [mh bacteremia]

#2 [mh sepsis]

#3 ("bloodstream" NEXT infection*):ti,ab

#4 Bacteremia:ti,ab

#5 Sepsis:ti,ab

#6 pyemia:ti,ab

#7 #1 OR #2 OR #3 OR #4 OR #5 OR #6

#8 [mh chills]

#9 [mh shivering]

#10 Shivering:ti,ab

#11 Chill*:ti,ab

#12 rigor:ti,ab

#13 shaking:ti,ab

#14 #8 OR #9 OR #10 OR #11 OR #12 OR #13

#15 #7 AND #14

#16 ([mh animals]) NOT ([mh humans])

#17 #15 NOT #16

Hits = 5 (16/11/2022)

#1 [mh bacteremia]

#2 [mh sepsis]

#3 ("bloodstream" NEXT infection*):ti,ab

#4 Bacteremia:ti,ab

#5 Sepsis:ti,ab

#6 pyemia:ti,ab

#7 #1 OR #2 OR #3 OR #4 OR #5 OR #6

#8 [mh chills]

#9 [mh shivering]

#10 Shivering:ti,ab

#11 Chill*:ti,ab

#12 rigor:ti,ab

#13 shaking:ti,ab

#14 #8 OR #9 OR #10 OR #11 OR #12 OR #13

#15 #7 AND #14

#16 ([mh animals]) NOT ([mh humans])

#17 #15 NOT #16

Hits = 7 (15/02/2024)

**MEDLINE (via PubMed) search strategy**

#1 bacteremia[MeSH Terms]

#2 sepsis[MeSH Terms]

#3 bloodstream infection*[Title/Abstract]

#4 Bacteremia[Title/Abstract]

#5 Sepsis[Title/Abstract]

#6 pyemia[Title/Abstract]

#7 #1 OR #2 OR #3 OR #4 OR #5 OR #6

#8 chills[MeSH Terms]

#9 shivering[MeSH Terms]

#10 Shivering[Title/Abstract]

#11 Chill*[Title/Abstract]

#12 rigor[Title/Abstract]

#13 shaking[Title/Abstract]

#14 #8 OR #9 OR #10 OR #11 OR #12 OR #13

#15 #7 AND #14

#16 ([mh animals]) NOT ([mh humans])

#17 #15 NOT #16

Hits = 613 (27/10/2021)

#1 bacteremia[MeSH Terms]

#2 sepsis[MeSH Terms]

#3 bloodstream infection*[Title/Abstract]

#4 Bacteremia[Title/Abstract]

#5 Sepsis[Title/Abstract]

#6 pyemia[Title/Abstract]

#7 #1 OR #2 OR #3 OR #4 OR #5 OR #6

#8 chills[MeSH Terms]

#9 shivering[MeSH Terms]

#10 Shivering[Title/Abstract]

#11 Chill*[Title/Abstract]

#12 rigor[Title/Abstract]

#13 shaking[Title/Abstract]

#14 #8 OR #9 OR #10 OR #11 OR #12 OR #13

#15 #7 AND #14

#16 ([mh animals]) NOT ([mh humans])

#17 #15 NOT #16

#18 ("2021/10/27"[Date - Publication] : "3000"[Date - Publication])

#19 #17 AND #18

Hits = 53 (17/11/2022)

#1 bacteremia[MeSH Terms]

#2 sepsis[MeSH Terms]

#3 bloodstream infection*[Title/Abstract]

#4 Bacteremia[Title/Abstract]

#5 Sepsis[Title/Abstract]

#6 pyemia[Title/Abstract]

#7 #1 OR #2 OR #3 OR #4 OR #5 OR #6

#8 chills[MeSH Terms]

#9 shivering[MeSH Terms]

#10 Shivering[Title/Abstract]

#11 Chill*[Title/Abstract]

#12 rigor[Title/Abstract]

#13 shaking[Title/Abstract]

#14 #8 OR #9 OR #10 OR #11 OR #12 OR #13

#15 #7 AND #14

#16 ([mh animals]) NOT ([mh humans])

#17 #15 NOT #16

#18 ("2022/11/17"[Date - Publication] : "3000/01/01"[Date - Publication])

#19 ("1700/01/01"[Date - Publication] : "2022/11/17"[Date - Publication])

#20　#18 NOT #19

#21　#17 AND #20

Hits = 62 (14/02/2024)

**EMBASE search strategy**

S1 EMB.EXACT.EXPLODE(“bacteremia”)

S2 EMB.EXACT.EXPLODE(“sepsis”)

S3 ab(bloodstream infection*) OR ti(bloodstream infection*)

S4 ab(Bacteremia) OR ti(Bacteremia)

S5 ab(Sepsis) OR ti(Sepsis)

S6 ab(pyemia) OR ti(pyemia)

S7 S1 OR S2 OR S3 OR S4 OR S5 OR S6

S8 EMB.EXACT.EXPLODE(“chill”)

S9 EMB.EXACT.EXPLODE(“Shivering”)

S10 ab(Shivering) OR ti(Shivering)

S11 ab(Chill*) OR ti(Chill*)

S12 ab(rigor) OR ti(rigor)

S13 ab(shaking) OR ti(shaking)

S14 S8 OR S9 OR S10 OR S11 OR S12 OR S13

S15 S7 AND S14

S16 EMB.EXACT.EXPLODE(“animal”) NOT EMB.EXACT.EXPLODE(“human”)

S17 S15 NOT S16

Hits = 3926 (20/10/2021)

S1 EMB.EXACT.EXPLODE(“bacteremia”)

S2 EMB.EXACT.EXPLODE(“sepsis”)

S3 ab(bloodstream infection*) OR ti(bloodstream infection*)

S4 ab(Bacteremia) OR ti(Bacteremia)

S5 ab(Sepsis) OR ti(Sepsis)

S6 ab(pyemia) OR ti(pyemia)

S7 S1 OR S2 OR S3 OR S4 OR S5 OR S6

S8 EMB.EXACT.EXPLODE(“chill”)

S9 EMB.EXACT.EXPLODE(“Shivering”)

S10 ab(Shivering) OR ti(Shivering)

S11 ab(Chill*) OR ti(Chill*)

S12 ab(rigor) OR ti(rigor)

S13 ab(shaking) OR ti(shaking)

S14 S8 OR S9 OR S10 OR S11 OR S12 OR S13

S15 S7 AND S14

S16 EMB.EXACT.EXPLODE(“animal”) NOT EMB.EXACT.EXPLODE(“human”)

S17 S15 NOT S16

S18 S17 AND pd(20211020-20221231)

Hits = 411 (16/11/2022)

S1 EMB.EXACT.EXPLODE(“bacteremia”)

S2 EMB.EXACT.EXPLODE(“sepsis”)

S3 ab(bloodstream infection*) OR ti(bloodstream infection*)

S4 ab(Bacteremia) OR ti(Bacteremia)

S5 ab(Sepsis) OR ti(Sepsis)

S6 ab(pyemia) OR ti(pyemia)

S7 S1 OR S2 OR S3 OR S4 OR S5 OR S6

S8 EMB.EXACT.EXPLODE(“chill”)

S9 EMB.EXACT.EXPLODE(“Shivering”)

S10 ab(Shivering) OR ti(Shivering)

S11 ab(Chill*) OR ti(Chill*)

S12 ab(rigor) OR ti(rigor)

S13 ab(shaking) OR ti(shaking)

S14 S8 OR S9 OR S10 OR S11 OR S12 OR S13

S15 S7 AND S14

S16 EMB.EXACT.EXPLODE(“animal”) NOT EMB.EXACT.EXPLODE(“human”)

S17 S15 NOT S16

S18 S17 and pd(20221116-30000101)

Hits = 525 (15/02/2024)

**ICTRP search strategy**

#1 Conditions: ("Bacteremia" OR "Sepsis" OR "bloodstream infection*" OR "pyemia")

#2 Intervention: ("Chill*" OR "Shivering" OR "rigor" OR "shaking")

#3 #1 AND #2

Recruitment status is ALL.

Hits = 0 (20/10/2021)

#1 Conditions: ("Bacteremia" OR "Sepsis" OR "bloodstream infection*" OR "pyemia")

#2 Intervention: ("Chill*" OR "Shivering" OR "rigor" OR "shaking")

#3 #1 AND #2

Recruitment status is ALL.

Hits = 0 (16/11/2022)

#1 Conditions: ("Bacteremia" OR "Sepsis" OR "bloodstream infection*" OR "pyemia")

#2 Intervention: ("Chill*" OR "Shivering" OR "rigor" OR "shaking")

#3 #1 AND #2

Recruitment status is ALL.

Hits = 0 (14/02/2024)

**ClinicalTrials.gov search strategy**

Condition or disease: "Bacteremia" OR "Sepsis" OR "bloodstream infection*" OR "pyemia"

Intervention: "Chill*" OR "Shivering" OR "rigor" OR “shaking”

Hits = 3 (20/10/2021)

Condition or disease: "Bacteremia" OR "Sepsis" OR "bloodstream infection*" OR "pyemia"

Intervention: "Chill*" OR "Shivering" OR "rigor" OR “shaking”

Hits = 0 (16/11/2022)

Condition or disease: "Bacteremia" OR "Sepsis" OR "bloodstream infection*" OR "pyemia"

Intervention: "Chill*" OR "Shivering" OR "rigor" OR “shaking”

Hits = 0 (14/02/2024)
