# Supplementary material for: Utility of shaking chills as a diagnostic sign for bacteremia in adults: a systematic review and meta-analysis
Source: BMC Med. 2024 Jun 11;22:240. doi: 10.1186/s12916-024-03467-z (PMC11167933; doi:10.1186/s12916-024-03467-z)
Supplement: Supplementary file 3 — Additional file 3: Table S1. Other characteristics of the included studies investigating shaking chills. ND, no data; APACHE, acute physiology and chronic health evaluation; SOFA, sequential organ failure assessment; SAPS, simplified acute physiology score; DM, diabetes mellitus; CKD, chronic kidney disease; HD, hemodialysis. *Two cohorts are presented separately as they were included in a study conducted by Sasaki in 2021. Table S2. Characteristics of the included studies investigating chills*. ND, no data; ED, emergency department; UTI, urinary tract infection; CAP, community-acquired pneumonia; ICU, intensive care unit; HIV, human immunodeficiency virus; AIDS, acquired immunodeficiency syndrome; CKD, chronic kidney disease; ESRD, end-stage renal disease; DM, diabetes mellitus; HD, hemodialysis; FUO, fever of unknown origin; APACHE, acute physiology and chronic health evaluation; SOFA, sequential organ failure assessment; PSI, pneumonia severity index; NEWS, national early warning score. *Information on the definition of contamination, the number of patients for whom contamination was detected, and the approach taken to analyze data of patients with contamination in their blood cultures were handled in each study. †If the original articles did not report the standard deviation for age, the age range was specified. [file 12916_2024_3467_MOESM3_ESM.docx]

Additional file 3.

**Table S1.** Other characteristics of the included studies investigating shaking chills

| **Author (location)** | **Year** | **Incubation time of blood cultures** | **Administered antibiotics before taking blood cultures, n (%)** | **Severity of condition (e.g., APACHE Ⅱ or Ⅲ score, SOFA score** | **Comorbidity (e.g., DM, CKD, HD, malignancy, and taking corticosteroid or immunosuppressant), n (%)** |
| --- | --- | --- | --- | --- | --- |
| Bahagon (Israel, single-center) [18] | 2007 | 5 days | 132 (37.7) | ND | DM, CKD, immunosuppressive data were collected but were not shown |
| Bates (the US, multicenter) [19] | 1997 | ND | Not available | APACHE III: mean 0.68 (SD 0.24); SAPS II: mean 25.7 (SD 14.4) | ND |
| Bates (the US, single-center) [20] | 1990 | 7 days | 349 (34.7) | ND | ND |
| Chassagne (France, multicenter) [21] | 1996 | ND | Excluded if used within 48 hours | ND | DM 14 (5.4),  malignancy 30 (11.6) |
| Fujii (Japan, multicenter) [4] | 2022 | 7 days | Not available | ND | ND |
| Holmqvist (Sweden, Switzerland, Canada, multicenter) [22] | 2020 | 5–21 days | 31 (15.7) | ICU admittance within 72 hours: 20 (10.2%) | DM 45 (22.8), CKD 39 (19.8), malignancy 38 (19.2), immunodeficiency 9 (4.6) |
| Hoogendoorn (Dutch, multicenter) [23] | 2002 | ND | 109 (14.3) | ND | ND |
| Komatsu (Japan, multicenter) [24] | 2017 | ND | 489 (26.5) | ND | ND |
| Lee (Taiwan, single-center) [7] | 2012 | 5 days | Not available | SAPS II >30 points:118 (29.8%) | DM 100 (25.3), malignancy 36 (9.1), renal insufficiency 45 (11.4) |
| McNab (Australia, single-center) [25] | 2023 | ND | Not available | ND | Immunodeficiency 372 (20) |
| Pfitzenmeyer (Switzerland, single-center) [26] | 1995 | 7 days | Not available | ND | DM 79 (14.2), malignancy 81 (14.5) |
| Sasaki (A)* (Japan, multicenter) [27] | 2021 | ND | 20 (5.6) | ND | DM 178 (49.4), HD 360 (100) |
| Sasaki (B)* (Japan, multicenter) [27] | 2021 | ND | 12 (12.5) | ND | DM 35 (36.5), HD 96 (100) |
| Sasaki (Japan, multicenter) [28] | 2017 | ND | 48 (16.4) | ND | DM 131 (44.7), HD 293 (100), malignancy 33 (11.3) |
| Takada (Japan, multicenter) [29] | 2021 | 7 days | Not available | ND | DM 439 (21.9) |
| Takamatsu (Japan, single-center) [30] | 2016 | ND | Not available | ND | ND |
| Taniguchi (Japan. Single-center) [9] | 2013 | ND | 70 (19.1) | ND | ND |
| Taniguchi (Japan, single-center) [31] | 2022 | 5 days | 40 (18.1) | Severe infection (septic shock, meningoencephalitis, requirement for debridement, or intensive care): 6 (0.03%) | DM 46 (20.8), CKD 7 (3.2), HD 3 (1.4), malignancy 34 (15.4), steroid use 8 (3.6), immunosuppressant use 2 (0.9) |
| Tokuda (Japan, single-center) [8] | 2005 | 7 days | 67 (12.7) | ND | Malignancy 32 (6.1) |
| Yoshino (Japan, single-center) [32] | 2023 | 5 days | Not available | ND | Hematological malignancy 471 (100) |

ND, no data; APACHE, acute physiology and chronic health evaluation; SOFA, sequential organ failure assessment; SAPS, simplified acute physiology score; DM, diabetes mellitus; CKD, chronic kidney disease; HD, hemodialysis

*Two cohorts are presented separately as they were included in a study conducted by Sasaki in 2021.

**Table S2.** Characteristics of the included studies investigating chills*

| **Author (location)** | **Year** | **Design** | **Enrollment** | **Inclusion criteria** | **Exclusion criteria** | **Number of analyzed patients** | **Age, years (mean, SD) †** | **Index test** | **Reference standard (n of sets)** | **Handling of contamination in blood culture (definition, n of patients, and analysis)** |
| --- | --- | --- | --- | --- | --- | --- | --- | --- | --- | --- |
| Choi (Korea, multicenter) [33] | 2023 | Retrospective | Consecutive | Patients aged ≥18 years who had at least two sets of blood cultures taken in the ED | Without matching ED physician notes | 15,362 | 62 (16) | Chills | Blood cultures (≥2) | No, 233, included |
| Falguera (Spain, multicenter) [34] | 2009 | Retrospective | Consecutive | Patients aged ≥18 years hospitalized for CAP | Tuberculosis, fungal infection, HIV, hematologic malignancy, solid-organ and bone marrow transplantation, neutropenia, treatment with immunosuppressive drugs, and previous antibiotic therapy | 1,386 | 63 (18) | Chills | Blood cultures (≥2) | Yes, 148, included |
| Fontanarosa (the US, single-center) [35] | 1992 | Retrospective | Case–control | Patients aged ≥ 65 years who underwent blood cultures because of suspected infection while in the ED and were hospitalized | Contamination of blood cultures | 215 | 79 (range 65–98) | Chills | Blood cultures (2) | Yes, 68, excluded |
| Fukui (Japan, single-center) [36] | 2022 | Retrospective | Consecutive | Patients who were diagnosed with pyelonephritis | Previous antibiotic therapy, obstructive pyelonephritis, and undergoing emergent stenting | 198 | 75 (15) | Chills | Blood cultures (unclear) | No, unclear, included |
| Hodgson (the UK, single-center) [37] | 2016 | Retrospective | Case–control | Patients aged ≥18 years who were hospitalized for at least 1 day | Hospital stay <1 night or admission to non-medical wards | 200 | 66 (17) | Chills | Blood cultures (unclear) | Yes, 50, excluded |
| Jaimes (Colombia, single-center) [38] | 2004 | Prospective | Consecutive | Patients aged >14 years who underwent blood cultures in adult services and were hospitalized | Pregnancy, history of organ transplantation, incomplete or unavailable clinical records, and death or discharge <24 hours after the initial blood culture | 500 | 45 (20) | Chills | Blood cultures (≥2) | Yes, 0, included |
| Jessen (Denmark, single-center) [39] | 2016 | Retrospective | Case–control | Patients aged >18 years who underwent blood cultures while in the ED | ND | 420 | 65 (20) | Chills | Blood cultures (≥1) | Yes, 61, included |
| Kim (Korea, single-center) [40] | 2011 | Prospective | Consecutive | Female patients aged ≥15 years with acute pyelonephritis who visited the ED | Severe sepsis or septic shock, immunocompromised state, CKD, acute renal failure, obstructive pyelonephritis, neurogenic bladder, indwelling urinary catheters, history of kidney transplantation, advanced age, liver cirrhosis, diabetes mellitus | 494 | 51 (19) | Chills | Blood cultures (≥2) | Yes, 41, included |
| Kuruoglu (Turkiye, single-center) [41] | 2023 | Retrospective | Case–control | Patients aged ≥18 years with Brucella Standard Tube Agglutination Test or Brucella Coombs Gel Test titer ≥ 1/160 and those with blood cultures which revealed Brucella spp | False positive results, insufficient data, treatment in another medical center, pregnancy, and organ involvement that could not be distinguished from comorbid diseases | 220 | 46 (16) | Sweat/Chills | Blood cultures (unclear) | No, unclear, excluded |
| Leibovici (Israel, single-center) [42] | 1991 | Prospective | Consecutive | Patients who were admitted to the Internal Medicine ward for fever of <2 weeks | ND | 244 | 71 (range 18–98) | Chills | Blood cultures (2) | Yes, unclear, included |
| Nimitvilai (Thailand, single-center) [43] | 2016 | Prospective | ND | Patients aged ≥18 years with fever, at least 1 symptom of UTI, and pyuria | Healthcare-associated infection, taking antibiotics for 72 hours before hospitalization, severe sepsis/septic shock | 106 (analyzed) | 44 (17) | Chills | Blood cultures (unclear) | No, 106, included |
| Phungoen (Thailand, single-center) [44] | 2021 | Retrospective | Consecutive | Patients aged >18 years with suspected infections for which blood cultures were performed and intravenous antibiotics were administered in the ED | Cardiac arrest, trauma, referral from other hospitals, previous antibiotic therapy, missing clinical data | 12,556 | 62 (range 18–100) | Chills | Blood cultures (2 aerobic bottles) | Yes, 0, included |
| Ratzinger (Austria, single-center) [45] | 2015 | Prospective | Consecutive | Patients aged ≥18 years in medical and surgical wards with suspected sepsis who underwent blood cultures | Inability to give consent, HIV infection, post-surgical care | 298 | 56 (17) | Chills | Blood cultures (unclear) | Yes, 6, excluded |
| Singh (the US, single-center) [46] | 2000 | Prospective | Consecutive | Liver transplant recipients with infections at liver transplant service | ND | 59 | 51 (range 25–73) | Chills | Blood cultures (unclear) | Yes, unclear, included |
| Su (Taiwan, single-center) [47] | 2011 | Prospective | Consecutive | Patients aged ≥15 years who underwent blood cultures while in the ED | Referral from other hospitals, previous antibiotic therapy, active thyroid cancer | 558 | 61 (19) | Chills | Blood cultures (≥2) | Yes, unclear, included |
| Takeshima (Japan, multicenter) [5] | 2016 | Retrospective | Consecutive | Patients aged ≥16 years who underwent blood cultures while in the ED | Only one sample for blood culture | 1,570 | 70 (19) | Chills | Blood cultures (≥2) | Yes, 109, included |
| Tromp (Netherlands, single-center) [48] | 2012 | Prospective | Consecutive | Patients aged ≥16 years who visited the ED for a suspected infection and had clinical signs of sepsis and hyperglycemia without diabetes mellitus | Missing additional laboratory marker data | 394 | 59 (20) | Chills | Blood cultures (2) | Yes, unclear, included |
| van Werkhoven (Dutch, muticenter) [49] | 2015 | Prospective | Consecutive | Patients aged ≥18 years with clinically suspected CAP and initially admitted to a non-ICU ward | ND | 2,977 | 68 (15) | Chills | Blood cultures (unclear) | Yes, 91, included |
| Xu (China, single-center) [50] | 2022 | Retrospective | Consecutive | Patients aged ≥18 years satisfying the criteria for classic FUO | Pregnancy; readmission within 3 months after discharge; discharge from hospital before identifying FUO etiology; or missing laboratory marker data | 712 | 52 (24) | Chills | Blood cultures (unclear) | Yes, unclear, included |
| Zhou (China, single-center) [51] | 2023 | Retrospective | Consecutive | Patients aged ≥18 years on maintenance HD who underwent blood cultures | Dialysis duration of less than two weeks or with acute renal failure | 391 | 58 (15) | Chills | Blood cultures (unclear) | No, unclear, included |

(Continued table)

| **Author (location)** | **Year** | **Incubation time of blood cultures** | **Administered antibiotics before taking blood cultures, n (%)** | **Severity of condition (e.g., APACHE Ⅱ or Ⅲ score, SOFA score)** | **Comorbidity (e.g., DM, CKD, HD, malignancy, and taking corticosteroid or immunosuppressant), n (%)** |
| --- | --- | --- | --- | --- | --- |
| Choi (Korea, multicenter) [33] | 2023 | Not available | Not available | ND | ND |
| Falguera (Spain, multicenter) [34] | 2009 | Not available | 359 (26) | PSI class IV: 458 (33%), PSI class V: 155 (11%) | DM 252 (18), CKD 50 (4), malignancy 98 (7) |
| Fontanarosa (the US, single-center) [35] | 1992 | Not available | 39 (18) | ND | DM 41 (19), malignancy 29 (14), steroid use 10 (5) |
| Fukui (Japan, single-center) [36] | 2022 | Not available | Excluded | qSOFA 2 points: 27/175 (15%), qSOFA 3 points: 3/175 (2%) (total number of the patients 198) | DM 51 (26), malignancy 42 (21), immunosuppressant use 16 (8) |
| Hodgson (the UK, single-center) [37] | 2016 | 5 days | Not available | ICU adimission: 11(6%) | DM 36(18), CKD 49 (25), malignancy 50 (25), steroid use 27 (14), Immunosuppressed 48(24), recent chemotherapy 20 (10) |
| Jaimes (Colombia, single-center) [38] | 2004 | Not available | 255 (51) | ND | Presence of comorbidity (HIV infection, chronic renal failure, diabetes mellitus, immunosuppressant chemotherapy, steroid use, and/or malignancy) 191 (38) |
| Jessen (Denmark, single-center) [39] | 2016 | 5.7 days | Not available | ND | DM 53 (13), malignancy 57 (14) |
| Kim (Korea, single-center) [40] | 2011 | Not available | 34 (7) | ND | DM 77 (16) |
| Kuruoglu (Turkiye, single-center) [41] | 2023 | Not available | Not available | ND | ND |
| Leibovici (Israel, single-center) [42] | 1991 | 7days | Not available | ND | DM 35 (14), renal failure 24 (10), solid organ malignancy 53 (22) |
| Nimitvilai (Thailand, single-center) [43] | 2016 | Not available | Excluded | ND | DM 22 (21) |
| Phungoen (Thailand, single-center) [44] | 2021 | ND | Excluded | qSOFA ≥2 points: 1,230 (15%); NEWS ≥7: 2,917 (36%) | DM 1,729 (21), moderate to severe CKD 639 (8), solid organ malignancy 1,878 (23), AIDS 123 (2) |
| Ratzinger (Austria, single-center) [45] | 2015 | Not available | 56 (19) | ICU stay: 13 (4%) | DM 46 (15), HD 18 (6), malignancy 124 (42) |
| Singh (the US, single-center) [46] | 2000 | Not available | Not available | APACHE II score: bacteremia group mean 17.4, non-bacteremia group mean 13.3 | DM 19 (37), HD 10 (19), steroid use 9 (17) |
| Su (Taiwan, single-center) [47] | 2011 | Not available | Not available | ND | DM 127(23), ESRD 16 (3), hematological malignancy 18 (3), solid organ malignancy 97 (17) |
| Takeshima (Japan, multicenter) [5] | 2016 | Not available | Not available | ND | DM 251 (17), HD 41 (3), malignancy 284 (19) |
| Tromp (Netherlands, single-center) [48] | 2012 | Not available | Not available | ICU stay: 20 (6%) | DM excluded |
| van Werkhoven (Dutch, muticenter) [49] | 2015 | Not available | 934 (31) | ND | DM 508 (17), renal failure 26 (0.9), malignancy 407 (14), Immunocompromised 582 (20) |
| Xu (China, single-center) [50] | 2022 | Within 48 hr of admission | Not available | ND | DM 72 (10.1), CKD 14 (2), solid tumor 24 (3.4), hematological malignancy 7 (1.0), steroid use 17 (2.4), chemotherapy 9 (1.3) |
| Zhou (China, single-center) [51] | 2023 | Not available | 94 (24) | ND | DM 121 (31), HD 391 (100), malignancy: 29 (7), steroid use 43 (11), immunosuppressant use 32 (8.2) |

ND, no data; ED, emergency department; UTI, urinary tract infection; CAP, community-acquired pneumonia; ICU, intensive care unit; HIV, human immunodeficiency virus; AIDS, acquired immunodeficiency syndrome; CKD, chronic kidney disease; ESRD, end-stage renal disease; DM, diabetes mellitus; HD, hemodialysis; FUO, fever of unknown origin; APACHE, acute physiology and chronic health evaluation; SOFA, sequential organ failure assessment; PSI, pneumonia severity index; NEWS, national early warning score

*Information on the definition of contamination, the number of patients for whom contamination was detected, and the approach taken to analyze data of patients with contamination in their blood cultures were handled in each study.

†If the original articles did not report the standard deviation of age, the age range was specified.

.
