# Supplementary material for: Utility of shaking chills as a diagnostic sign for bacteremia in adults: a systematic review and meta-analysis
Source: BMC Med. 2024 Jun 11;22:240. doi: 10.1186/s12916-024-03467-z (PMC11167933; doi:10.1186/s12916-024-03467-z)
Supplement: Supplementary file 4 — Additional file 4: Fig. S1. Risk-of-bias assessment of the included studies for all chills using the QUADAS-2 tool*. *Two cohorts are presented separately as they were included in a study conducted by Sasaki in 2021. Fig. S2. Summary of the QUADAS-2 risk-of-bias assessments in included studies for all chills*. *Two cohorts were included in a study conducted by Sasaki in 2021. This accounts for the total of 40 studies in this figure, although 39 studies were incorporated in our review. [file 12916_2024_3467_MOESM4_ESM.docx]

Additional file 4

**Fig. S1.** Risk-of-bias assessment of the included studies for all chills using the QUADAS-2 tool *

*Two cohorts are presented separately as they were included in a study conducted by Sasaki in 2021.

**Fig. S2.** Summary of the QUADAS-2 risk-of-bias assessments in included studies for all chills *

*Two cohorts were included in a study conducted by Sasaki in 2021. This accounts for the total of 40 studies in this figure, although 39 studies were incorporated in our review.
