# Supplementary material for: Utility of shaking chills as a diagnostic sign for bacteremia in adults: a systematic review and meta-analysis
Source: BMC Med. 2024 Jun 11;22:240. doi: 10.1186/s12916-024-03467-z (PMC11167933; doi:10.1186/s12916-024-03467-z)
Supplement: Supplementary file 5 — Additional file 5: Fig. S3. Forest plot of all the included studies using all chills as the index test. Fig. S4. HSROC analysis based on the bivariate model of all the included studies using all chills. HSROC, hierarchical summary receiver operating characteristic. [file 12916_2024_3467_MOESM5_ESM.docx]

Additional file 5

**Fig. S3.** Forest plot of all the included studies using all chills as the index test

**Fig. S4.** HSROC analysis based on the bivariate model of all the included studies using all chills

HSROC, hierarchical summary receiver operating characteristic.
