# Supplementary material for: Utility of shaking chills as a diagnostic sign for bacteremia in adults: a systematic review and meta-analysis
Source: BMC Med. 2024 Jun 11;22:240. doi: 10.1186/s12916-024-03467-z (PMC11167933; doi:10.1186/s12916-024-03467-z)
Supplement: Supplementary file 6 — Additional file 6: Fig. S5. Forest plot of studies analyzed for patients with suspected bacteremia, including those with contaminated blood cultures. Fig. S6. HSROC analysis based on the bivariate model of studies analyzed for patients with suspected bacteremia, including those with contaminated blood cultures. HSROC, hierarchical summary receiver operating characteristic. [file 12916_2024_3467_MOESM6_ESM.docx]

Additional file 6

**Fig. S5.** Forest plot of studies analyzed for patients with suspected bacteremia, including those with contaminated blood cultures

**Fig. S6.** HSROC analysis based on the bivariate model of studies analyzed for patients with suspected bacteremia, including those with contaminated blood cultures

HSROC, hierarchical summary receiver operating characteristic.
